# Ovarian cancer with intestinal wall invasion and hyperamylasemia: a case report

**DOI:** 10.3389/fonc.2024.1299226

**Published:** 2024-02-09

**Authors:** Yu Jie, Juan Li, Chang-feng Man, Yu Fan

**Affiliations:** Cancer Institute, The Affiliated People’s Hospital of Jiangsu University, Zhenjiang, Jiangsu, China

**Keywords:** ovarian cancer, hyperamylasemia, neoplasm metastasis, diagnosis, case reports

## Abstract

Numerous studies have suggested a robust association between amylase and ovarian cancer. however, few amylase-producing ovarian cancers have been reported because amylase is a rare product of ovarian cancer. A case of an elderly female patient with an upper abdominal unfitness, intestinal wall along with uterine adnexal invasion, and high serum and urinary amylase is summarized in this article. The patient was initially suspected of having a gastrointestinal tumor. Initial laboratory findings showed markedly significantly raised serum and urinary amylase levels. Imaging showed invasion of the intestinal wall and uterine adnexa, and histology of the specimen taken through the abdominal wall lump and electron colonoscopy showed ovarian cancer. The patient’s blood amylase levels decreased to normal after 4 cycles of neoadjuvant chemotherapy with paclitaxel and carboplatin. Following this, she underwent interval debulking surgery, which included total hysterectomy, bilateral adnexectomy, great omentectomy, appendectomy, resection of pelvic and abdominal lesions, and partial rectal resection. Postoperative pathology and immunohistochemistry staining confirmed a diagnosis of high-grade serous ovarian cancer. This case suggests that in female patients, hyperamylasemia may indicate the presence of ovarian cancer. It is necessary to perform a multisite, multipoint histologic examination to identify the tumor’s origin in patients with multiple sites of invasion.

## 1 Introduction

Ovarian cancer is one of the three most common malignant tumors of the female genital system, which cannot be easily diagnosed at an early stage due to inconspicuous clinical symptoms (1). Serum amylase, one of the digestive enzymes, can be elevated in a variety of disease states, including pancreatic inflammation, pancreatic cancer, and other malignant tumors (2–4). In recent years, many studies have been dedicated to exploring the application of serum enzyme profiles in ovarian cancer (5, 6), with particular focus on the role of serum amylase in the diagnosis and prognostic evaluation of ovarian cancer (7).

We summarized a case of an ovarian cancer patient who had intestinal wall invasion along with high Serum and urinary amylase, and performed a literature review of amylase elevation arising from ovarian cancer. We encourage clinicians to consider ovarian cancer as one of the possible diagnoses of hyperamylasemia in female patients.

## 2 Case report

**Figure 1:**
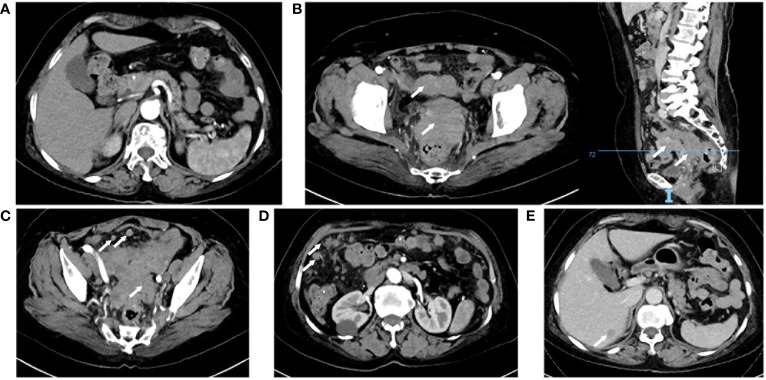
Computed tomography enhanced scan. **(A)** Normal pancreas. **(B)** Uterine adnexal mass and pelvic metastatic lesion, right: sagittal view. **(C)** Thickening of the junction area between the rectum and the sigmoid colon and multiple pelvic nodules. **(D)** Multiple nodules in the abdominal cavity. **(E)** Venous phase, a nodule in the right lobe of the liver.

A 66-year-old woman was admitted to the oncology clinic with epigastric discomfort for more than a month. Her symptoms mainly consisted of epigastric fullness and discomfort in the abdomen, with occasional vague pain in the epigastric region, which could be relieved after feeding. She had no specific history or family history of tumors. The Patient’s initial gastroscopy at an outside hospital showed chronic superficial gastritis and duodenal bulbar inflammation, and she was poorly treated with acid-suppressing therapy. A computed tomography (CT) scan of the upper abdomen at the local hospital showed a slightly hypodense nodule in the right liver, thickened peritoneum, and multiple nodular and clumpy shadows. Subsequently, she presented to our outpatient clinic for consultation. Physical examination revealed abdominal distension with tenderness in the upper abdomen, but no palpable masses were detected. The outpatient doctor admitted her to the Gastrointestinal Oncology Unit of the Chemotherapy Department, suspecting a digestive tract tumor. After admission, the patient had no significant discomfort in the upper abdomen. However, the serum and urine amylase levels (**Table 1**), as well as serum carbohydrate antigen 125 (CA125) and carbohydrate antigen 72-4 (CA72-4) were distinctly elevated, while carcinoembryonic antigen (CEA) level was within the normal range. CT scans of the abdomen and pelvis (**Figure 1**) showed no pancreatic exudation, multiple hyperintense nodules in the abdominopelvic cavity and greater omental peritoneum, patchy dense shadows in the adnexal region of the pelvis, poorly displayed uterine region, thickened wall of the junction between the sigmoid colon and the rectum, and a nodule in the right lobe of the liver.

**Table 1:** Serum and urine amylase tests.

Testing time	Serum amylase (U/L)	Urinary amylase (U/L)
First day of hospitalization	1734(20~220)	7605(200~1000)
Second day of hospitalization	1619(20~220)	9993(200~1000)
First week of hospitalization	1584(20~220)	7423(200~1000)
After 1 cycle of chemotherapy	517(20~220)	1130(200~1000)
After 2 cycles of chemotherapy	173(20~220)	427(200~1000)
After 3 cycles of chemotherapy	141(20~220)	/
During the fourth cycle of chemotherapy	102(20~220)	/

**Figure 2:**
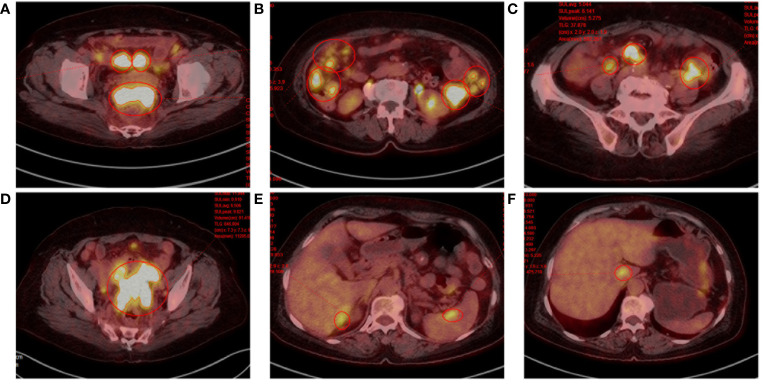
Positron emission tomography. **(A)** Rectouterine pouch and uterine adnexal mass. **(B-D)** Multiple nodular shadows in the abdominopelvic cavity and greater omentum, inhomogeneous thickening of the local peritoneum adjacent to the left colon. **(E, F)** Hypodense shadow in the right lobe of the liver with increased FDG metabolism; foci of increased FDG metabolism in the caudate lobe of the liver and spleen.

**Figure 3:**
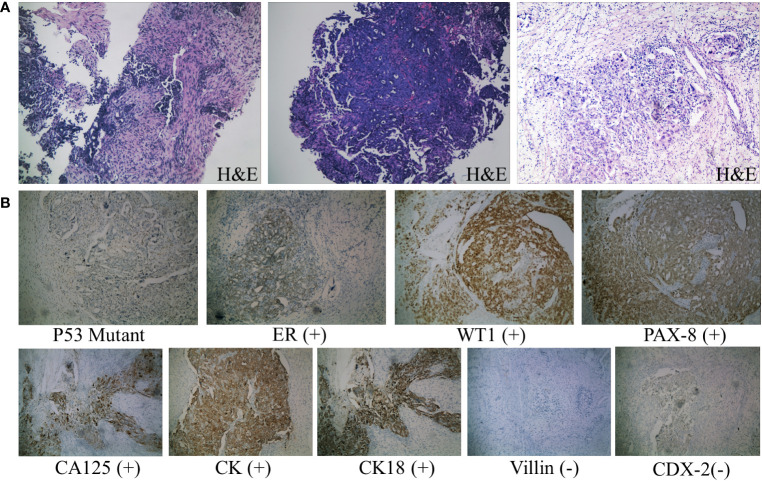
Pathological diagnosis. **(A)** From left to right: H&E staining results (×100) of needle biopsy of abdominal wall mass, endoscopic biopsy specimen, and surgical resection of ovarian tissue. The tumor cells are oval-shaped, varying in size, with large, deeply stained nuclei, prominent cell atypia, and easily observable mitotic figures. **(B)** Immunohistochemical staining results of ovarian tissue (×100). Consistent with the features of HGSOC according to the WHO classification: diffuse nuclear expression of WT1, mutant p53 expression, positive staining for CA125 and PAX8, and frequent ER expression. Negative staining for common immunohistochemical markers of colorectal cancer, such as Villin and CDX-2 proteins.

During the patient’s hospitalization, there were no significant abdominal pain symptoms. Based on the imaging examinations, we ruled out the possibility of acute pancreatitis. However, follow-up blood and urine amylase tests the next day showed no decrease. To define the origin of the tumor, the patient underwent Positron Emission Tomography (PET) which revealed invasions of the abdominal wall and intestinal wall (**Figure 2**). The patient then underwent percutaneous biopsy of the abdominal wall mass and endoscopic biopsy to obtain tissue samples for immunohistochemical staining. The histological examination of the percutaneous biopsy of the abdominal wall mass showed poorly differentiated carcinoma, suggesting a possible adnexal origin (**Figure 3A**). Immunohistochemical staining results indicated positivity for Cytokeratin 18 (CK18), P53 (weak), Cytokeratin (CK), Estrogen receptor (ER), Wilms’ tumor (WT-1), Paired box 8 (PAX-8), and CA125 proteins, while Progesterone receptor (PR), Villin, and CDX-2 were negative. Further endoscopic biopsy was performed, and the histological examination revealed poorly differentiated carcinoma, suggesting a possible adnexal origin (**Figure 3A**). The immunohistochemical results showed positivity for PAX-8, WT-1, ER, and CK proteins, while Villin, CDX2, PR, and Cytokeratin 20 (CK20) were negative. Nonsense mutation was detected in P53. According to the WHO classification (8), the preoperative histopathological examination and immunohistochemical staining results suggest a potential diagnosis of high-grade serous ovarian cancer (HGSOC). Considering the absence of significant gastrointestinal symptoms during the patient’s hospitalization, elevated CA125 levels, increased amylase levels, and the location of the tumor as indicated by imaging, the patient was diagnosed with advanced-stage ovarian cancer with implantation metastasis (stage IV).

According to the National Comprehensive Cancer Network (NCCN) guidelines (9), the patient received neoadjuvant chemotherapy with the specific regimen of paclitaxel 120mg and cisplatin 200mg administered by intravenous infusion on days 1 and 8, respectively. After three cycles of chemotherapy, her blood CA125 and amylase levels gradually decreased (**Table 1**), and a follow-up CT scan showed an improvement in the pelvic and abdominal lesions compared to before. Due to the complexity of the surgery, the patient underwent an additional cycle of the original chemotherapy regimen. Subsequently, she underwent interval debulking surgery, which involved the removal of the uterus, bilateral adnexa (fallopian tubes and ovaries), great omentum, appendix, pelvic and abdominal lesions, and partial rectum. The postoperative pathology report revealed the presence of cancerous tissue in the bilateral adnexa, pelvic floor, omentum majus, left colon, appendiceal fat tissue, and rectum. Immunohistochemistry staining was performed, and the results showed positive expression for CK18, CK, ER, P53 mutation type, WT1, PAX-8, and CA125, while PR, Villin, and CDX2 were negative. These findings further confirmed the diagnosis of HGSOC (**Figure 3B**). Following surgery, the patient had the original chemotherapy therapy schedule for two cycles. She is still under close follow-up with no signs of tumor progression. The patient has given informed consent to publish the case.

## 3 Discussion

Ovarian cancer is one of the most common malignant tumors in the female reproductive system (1). However, due to atypical early symptoms, such as abdominal distension and dyspepsia, it is frequently disregarded or confused with other diseases, resulting in missing the best time for treatment. There are two isoenzymes of amylase: the S-type (also known as salivary gland type amylase) and the P-type (also known as pancreatic amylase) (3). Previous studies have suggested an association between amylase and ovarian cancer (10), however, there have been few reports on ovarian cancer that produce amylase since amylase is a rare product of ovarian cancer. The first case of hyperamylasemia related to ovarian cancer was reported in 1975 (11), followed by several cases of amylase elevation arising from ovarian cancer (12–14).

Pathologically, ovarian cancers associated with elevated amylase levels are mostly serous carcinomas, and the increased amylase is of salivary type. However, the specific mechanism remains unclear. The fallopian tubes themselves can secrete amylase, and the epithelium of serous ovarian carcinoma shares similar tissue structure with the epithelium of the fallopian tube, which may be one of the reasons (15, 16). Some scholars have presented amylase isoenzymes as possible biomarkers for assessing the prognosis and efficacy of ovarian cancer. For example, Zakrzewska (17, 18) found that total amylase activity and its salivary isoforms in serum were decreased in patients with ovarian cancer after radiotherapy and surgery. Vuković (7) found remarkable elevation of amylase levels correlated with poorer survival in ovarian malignancies.

It is easy to mistake ovarian cancer with hyperamylasemia for acute pancreatitis. Acute pancreatitis is a common cause of increased blood amylase, and the elevated amylase it causes is predominantly pancreatic type. An episode of acute pancreatitis is characterized by acute, persistent upper and middle abdominal pain and imaging showing morphological changes in the pancreas. In this case, neither pancreatic exudation on CT nor the usual indications of abdominal pain were present in the patient. So excluding the diagnosis of acute pancreatitis, hyperamylasemia was considered to be caused by ovarian cancer. Given the lack of definitive basis, doctors were still monitoring the patient’s blood and urine amylase levels and paying attention to her abdominal signs during the course of treatment. The decrease in serum amylase to the normal range after chemotherapy further confirmed that the hyperamylasemia resulted from ovarian cancer and was consistent with previous studies of serum amylase as a marker for assessing the prognosis and efficacy of ovarian cancer (17, 18). Although elevated serum amylase levels cannot specifically indicate ovarian cancer, female patients with hyperamylasemia should be vigilant about the presence of ovarian cancer after common causes of elevated amylase have been excluded.

Although this is not the first reported case of ovarian cancer causing elevated blood amylase levels, our case is more unique. The patient did not exhibit the typical clinical signs of ovarian cancer, such as abdominal mass and ascites, and the imaging showed simultaneous invasion of the intestinal wall and uterine adnexa, which was readily mistaken as plantation metastasis of gastrointestinal tumor. Ovarian cancer commonly metastasizes to the pelvic and abdominal cavities, with the most common and serious site of metastasis being the intestinal tract. Additionally, about 10-25 percent of ovarian tumors are metastatic, mainly from the gastrointestinal tract (19). Identifying the primary tumor source is essential since it affects the best approach to treatment and prognosis for cancers. For cases presenting with simultaneous involvement of the intestinal wall and adnexa during initial diagnosis, it is difficult to clinically differentiate between ovarian and gastrointestinal sources. The comprehensive application of methods such as serum amylase, tumor markers, colonoscopy, histopathology, and immunohistochemistry can assist in differential diagnosis. Studies have demonstrated the significant value of the CA125/CEA ratio in differentiating between ovarian and non-ovarian tumors (20, 21). When the CA125/CEA ratio is greater than 25, it is important to be cautious about the presence of primary ovarian cancer. The combined use of immunohistochemical markers can provide better guidance in distinguishing primary ovarian cancer from secondary ovarian tumors of gastrointestinal origin (22, 23), such as CK20, Villin, CDX-2, CEA, CA125, etc. (24, 25). Typical secondary ovarian tumors of gastrointestinal origin present with positive staining for Villin, CDX-2, CK20, and CEA, while they are negative for CA125. In contrast, primary ovarian cancer often exhibits the opposite pattern of staining. After acute pancreatitis was ruled out in this individual, abnormally elevated blood amylase and CA125 indicated the likelihood of ovarian cancer. Therefore, we have implemented additional measures including PET, percutaneous biopsy of abdominal wall masses, specimen retrieval through electronic colonoscopy, and immunohistochemical staining. According to the WHO classification (8), based on the histopathology and immunohistochemical staining of preoperative biopsy samples, it is considered as HGSOC. According to the patient’s lack of obvious gastrointestinal symptoms during hospitalization, elevated levels of CA125 and amylase, tumor distribution shown by imaging, as well as the histopathological and immunohistochemical staining results of preoperative biopsy, the patient was diagnosed with primary ovarian cancer. For patients with malignant tumors involving multiple sites, it is necessary to conduct comprehensive PET-CT scans and histological examinations at multiple sites in order to accurately determine the primary tumor origin, especially to exclude multiple primary malignant tumors (MPMT). MPMT refer to the simultaneous or sequential detection of two or more primary malignant tumors in single or multiple organs of the same patient (26). MPMT are typically linked with worse malignant behavior and prognosis than a single primary tumor. The treatment of MPMT is also relatively complex and individualized as it addresses the decision of which tumor to treat initially. For multisite malignancies, clinicians should consider the possibility of multiple primary cancers existing. In this particular case, clinically, we cannot exclude the possibility of MPMT. Therefore, we conducted further comprehensive PET-CT scans and histological examinations at multiple sites. The results indicated that the malignancy originated from the ovaries. There are some limitations to this study. The specific type of elevated amylase in the serum was not identified through amylase isoenzyme electrophoresis during the diagnostic and treatment process. Additionally, the expression of salivary-type amylase in the tumor was not confirmed through immunohistochemical staining.

## 4 Conclusion

Hyperamylasemia is a rare manifestation of ovarian cancer, and we encourage clinicians to consider ovarian cancer as one of the possible diagnoses for female patients with elevated serum amylase after common causes of hyperamylasemia have been ruled out. Histomorphological examinations of multiple sites and points are required to eliminate the possibility of MPMT in patients with tumors that show multiple sites of invasion on imaging.

## Patient perspective

The patient expressed satisfaction with the outcome of the treatment and was motivated to undergo postoperative chemotherapy as scheduled.

## Data availability statement

The raw data supporting the conclusions of this article will be made available by the authors, without undue reservation.

## Ethics statement

The studies involving humans were approved by the Ethics Committee of the Affiliated People’s Hospital of Jiangsu University. The studies were conducted in accordance with the local legislation and institutional requirements. Written informed consent for participation was not required from the participants or the participants’ legal guardians/next of kin in accordance with the national legislation and institutional requirements. Written informed consent was obtained from the individual(s) for the publication of any potentially identifiable images or data included in this article.

## Author contributions

YJ: Data curation, Investigation, Supervision, Visualization, Writing – original draft, Writing – review & editing. JL: Conceptualization, Data curation, Investigation, Resources, Supervision, Validation, Writing – review & editing. C-FM: Data curation, Investigation, Resources, Supervision, Validation, Writing – review & editing. YF: Conceptualization, Investigation, Methodology, Resources, Supervision, Validation, Writing – review & editing.
